# Pharmacokinetic–Pharmacodynamic Modeling in Pediatric Drug Development, and the Importance of Standardized Scaling of Clearance

**DOI:** 10.1007/s40262-018-0659-0

**Published:** 2018-04-19

**Authors:** Eva Germovsek, Charlotte I. S. Barker, Mike Sharland, Joseph F. Standing

**Affiliations:** 1 Infection, Inflammation and Rheumatology Section, UCL Great Ormond Street Institute of Child Heath, University College London, London, UK; 2 Pharmacometrics Research Group, Department of Pharmaceutical Biosciences, Uppsala University, PO Box 591, 751 24 Uppsala, Sweden; 3 Paediatric Infectious Diseases Research Group, Institute for Infection and Immunity, St George’s, University of London, London, UK; 4 St George’s University Hospitals NHS Foundation Trust, London, UK

## Abstract

Pharmacokinetic/pharmacodynamic (PKPD) modeling is important in the design and conduct of clinical pharmacology research in children. During drug development, PKPD modeling and simulation should underpin rational trial design and facilitate extrapolation to investigate efficacy and safety. The application of PKPD modeling to optimize dosing recommendations and therapeutic drug monitoring is also increasing, and PKPD model-based dose individualization will become a core feature of personalized medicine. Following extensive progress on pediatric PK modeling, a greater emphasis now needs to be placed on PD modeling to understand age-related changes in drug effects. This paper discusses the principles of PKPD modeling in the context of pediatric drug development, summarizing how important PK parameters, such as clearance (CL), are scaled with size and age, and highlights a standardized method for CL scaling in children. One standard scaling method would facilitate comparison of PK parameters across multiple studies, thus increasing the utility of existing PK models and facilitating optimal design of new studies.

## Key Points

**Table:** 

Pharmacokinetic/pharmacodynamic (PKPD) modeling is important in the design and conduct of clinical pharmacology research in children, and the so-called ‘population’ approach is suitable for rich or sparse data in terms of the number of samples per subject
The utility of pediatric PK models can be increased by using a standardized approach to scaling: a suggested method for scaling clearance (CL) is a combination of allometric weight scaling with a sigmoid function to account for organ maturation. This should be used a priori, as a ‘base’ approach, allowing the effects of age and size to be delineated from other patient-specific factors, such as disease state and organ (dys)function
When determining the pediatric dose, instead of directly scaling the dose from adults to children, the pediatric PK parameter estimates should be obtained from a PK model with a standardized scaling approach in order to avoid the use of arbitrary cut-off values (of age/weight) according to a specific (non-standardized) CL-scaling formula
Significant progress has recently been made on pediatric PK modeling; a greater emphasis now needs to be placed on PD modeling to understand age-related changes in drug effects
PKPD model-based dose individualization is becoming increasingly popular as the age of personalized medicine dawns

## Introduction

During the evolution of modern drug development, pediatrics and neonatology were largely neglected, rendering children and infants ‘therapeutic orphans’ [1, 2]. However, in recent years there has been an increase in research activity to support the development of evidence-based pharmacotherapy for children stimulated by the advent of new legislation to mandate licensing for new medicinal products in this population [3, 4]. Pharmacokinetic/pharmacodynamic (PKPD) modeling and simulation (M&S) play a pivotal role in pediatric drug development through supporting rational trial design and increasingly replacing traditional trials through extrapolation of efficacy and safety [5–7]. Furthermore, the application of PKPD modeling to optimize dosing recommendations and therapeutic drug monitoring (TDM) strategies is also increasingly recognized. Historically, children were originally treated as ‘small adults’, i.e. the dose was simply scaled down per linear weight, leading potentially to overdosing in very small children, especially neonates, as their kidneys and liver were not yet fully developed, often resulting in slower drug elimination [8]. As awareness of developmental pharmacology subsequently expanded, the physiological differences in drug handling between children and adults were emphasized, leading to the notion that ‘children are not small adults’ [9]. However, rather than dichotomizing adult and pediatric patients, recognition that maturation is a continuous process has since led to acknowledgment of the need to quantify differences and understand similarities across the age range with appropriate scaling. This paper discusses the principles of PKPD modeling in the context of pediatric drug development, and highlights the importance and benefits of using one standardized method for scaling clearance (CL) in children.

## Pediatric Drug Development: Background and Legislation

In many regions, including the US and Europe, regulations requiring pharmaceutical companies to demonstrate both safety and efficacy of their products prior to marketing were introduced following the tragic events relating to thalidomide use during the 1960s [10]. Despite the fact that infants were the main victims of thalidomide’s teratogenic effects, the resultant drug legislation was not specifically targeted towards children or neonates [1]. This meant that unlicensed and off-label use of medicines has remained unavoidably commonplace in pediatrics and neonatology, together with its associated risks [11–14]. However, in recent years, specific legislation has come into effect that enshrines pediatric medicines research in law for new medicinal products licensed within the relevant jurisdictions [15, 16]. Some key landmarks in these developments are outlined in Table 1.

**Table 1:** Key landmarks in pediatric medicines regulation

Year	Regulation	Impact
1997	US FDA Modernization Act (FDAMA)	This act presented the financial incentive of an additional 6 months of market exclusivity to companies undertaking required pediatric studies [15]
1998	US FDA Pediatric Rule	This rule permitted companies to label medicines for use in children based on extrapolation of efficacy from adult trial data, together with pediatric PKPD and safety data [160]
2002 (and 2007)	US Best Pharmaceutical for Children Act (BPCA)	Framework for pediatric research in both on- and off-patent drugs [161]
2003	US Pediatric Research Equity Act (PREA)	Sponsors required to undertake clinical studies in children for new medicines and biological products [161]
2007	EU Pediatric Regulation	Introduction of new legislation in the European Union mandating pediatric medicines research for new medicinal products [16]
2012	US Food and Drug Administration Safety and Innovation Act (FDASIA)	BPCA and PREA became permanent in US Law [162]

*PKPD* pharmacokinetic/pharmacodynamic

In both the European Union (EU) and the US, the introduction of such legislation has necessitated the consideration of children and neonates earlier during the drug development process. During the last 20 years, the use of M&S to support pharmacological research in children, as well as adults, has also advanced significantly. The techniques of M&S may be applied in various contexts and are not solely limited to PKPD studies (where they are often known as pharmacometrics); these techniques are discussed further below. It is important to note that the applications and benefits of M&S in drug development and postmarketing drug research are often at risk of being underutilized [17], especially in circumstances when there may be limited expertise or if there is ineffective dialogue between those specialized in modeling (e.g. ‘pharmacometricians’ [18, 19]) and those directly involved in patient care, i.e. clinicians and pharmacists. Fortunately, the benefits of applying quantitative pharmacological methods in clinical practice are now more widely appreciated [20], in addition to their key roles in research and drug development [21–25]. To reflect this, there is also a growing number of experts in M&S within regulatory agencies [26–28].

### Extrapolating the Dose from Adults to Children

Before considering the pediatric population during drug development, PKPD data are normally first available from adult subjects, either healthy volunteers and/or patients. This then generates questions surrounding the extrapolation of PKPD information to children, regarding when one can appropriately use extrapolation techniques [29] and what information can be safely extrapolated.

Extrapolation can be defined as [abbreviated] “Extending information and conclusions available from studies in one or more subgroups of the patient population (source population) […] to make inferences for another subgroup of the population (target population), or condition or product, thus reducing the need to generate additional information […] to reach conclusions for the target population […]” [30].

Before undertaking extrapolation exercises, various distinct aspects of pharmacology must be considered, including the following.

1. Pharmacokinetics: Absorption, distribution, metabolism, elimination (ADME), and the influence of developmental pharmacology and ontogeny on the drug’s PK profile in children of different ages. The impact of comorbidities, e.g. renal failure [31], on drug disposition may also need to be evaluated, where relevant.
2. Pharmacodynamics (including both efficacy and toxicity): Consideration of host developmental and receptor pharmacology, which can affect both the desired drug effect, through the principal mode of action, and any off-target effects, which may be desirable (e.g. the anti-inflammatory activity of macrolide antibiotics [32]), neutral (i.e. of no clinically important consequence), or toxic. When the target receptor is in another organism, e.g. the drug target located within the pathogen in the case of anti-infective therapies, consideration must also be given as to whether the target pathogen’s resistance profile is likely to be the same in neonates and children as in adults.
3. Formulations: Pediatric age-appropriate, formulation-related issues should be addressed as early as possible [33], with consideration of a number of factors such as any potential for excipient-related toxicity [34, 35], and also palatability and the development of child-friendly dosage regimens where feasible, both of which will affect compliance in children [36–38].

M&S can contribute to each of the assessments outlined above; ranging from dose selection for neonatal/pediatric clinical studies [39], to investigating the impact of altered dosing regimens that would fit in better with a child’s daily routine. Thus, the appropriate use of M&S for extrapolation approaches is of particular significance in pediatric drug development [40].

**Fig. 1:**
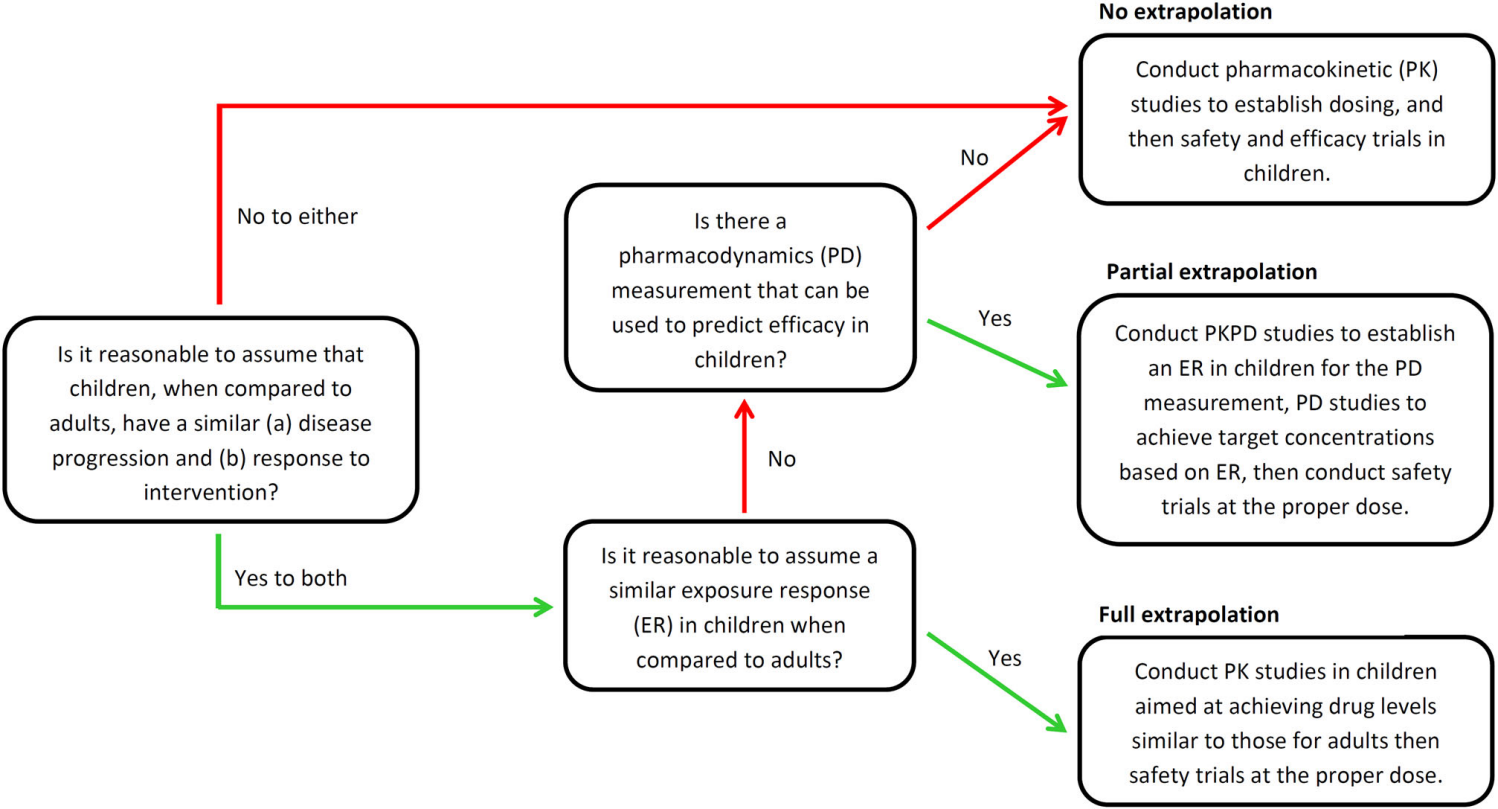
Decision tree for pediatric studies. Adapted from Dunne et al. [42]. *PKPD* pharmacokinetic/pharmacodynamic

For determining when extrapolation is or is not appropriate, a useful point of reference is the US FDA Pediatric Study Decision Tree, shown in Fig. 1 (adapted), which has been developed for supporting decision making regarding extrapolation during pediatric drug development [41, 42].

This decision tree highlights the need to evaluate different issues, including the following.

1. Natural history of disease progression in children/adults, and response to therapeutic intervention.
2. Likely exposure–response profile: Is it sufficient to simply target the same drug exposure (e.g. with respect to the area under the curve (AUC) in the PK profile, once this has been scaled appropriately; see further details below)?
3. Suitability of the pharmacodynamic measures and whether these are applicable to children: Is the PKPD index likely to be the same? For example, during antimicrobial therapy, typical PKPD indices, such as the peak concentration (*C*_max_) for aminoglycoside therapy, are used in both adult and pediatric populations [43, 44], but, for some medications/conditions (such as pulmonary hypertension in neonates [45]), different pediatric PD measures are required.

The science of extrapolation is a rapidly evolving area, with many aspects that warrant detailed research [42, 46–49]. In future, it is likely that there will be further guidance about related issues, such as the appropriateness of extrapolation of data between agents within the same therapeutic class, and how to extrapolate from in vivo models to neonates for conditions that do not have a counterpart within the adult population [50]. Overall, extrapolation should now be viewed as an ethical requirement for pediatric drug development since it can reduce the chance of undertaking unnecessary research and support the design of those pediatric studies that are required [42]. Accordingly, this important topic has been the focus of recent new regulatory documents from both the European Medicines Agency [51] and the International Council on Harmonization [52].

### Different Levels of Evidence Required

In different circumstances, varying levels of PKPD and safety data may be required, depending on the particular drug concerned and the availability of existing, relevant data in pediatric and neonatal populations. It is important to assess both the quantity and quality of the PKPD data that are available in particular pediatric populations [53]. There are a number of regulatory guidelines that provide advice to pharmaceutical companies on these topics (for example, see [53–58]). Some key issues can remain in the ‘gray’ areas; for example, the best way to do sample size calculations for pediatric PKPD studies that will use population modeling for the analysis. Previously, Tam et al. investigated the sample size required to generate robust PK predictions when using population modeling with Monte Carlo simulations to predict antimicrobial PK variability [59]. To obtain reasonably robust predictions, it was recommended that a non-parametric model derived from a sample population size of at least 50 subjects was needed as the input information [59]. However, limited data are currently available to support evidence-based target setting within specific subpopulations, such as neonates born at varying stages of prematurity and with varying degrees of organ dysfunction. Early consultation with pediatric or neonatal clinical pharmacologists and pharmacists is advisable during the protocol development stage to address these challenges. Pragmatic considerations are also important to ensure the feasibility of study targets, with consideration of whether they are realistically achievable within the desired time frames, as recruitment rates into pediatric trials (including simple PK studies) are often surprisingly low [60, 61]. The FDA have issued specific guidance on pediatric PK study sample size calculation as follows: “The study must be prospectively powered to target a 95% confidence interval (CI) within 60 and 140% of the geometric mean estimates of clearance and volume of distribution for DRUG NAME in each pediatric sub-group with at least 80% power” [62], and Stockmann et al. published recommendations on how to undertake such calculations [63]. It is important to recognize that such sample size calculations will inevitably be affected by uncertainty of the expected pharmacokinetic parameters in the target population, and therefore adaptive optimal designs may have a role to play in target refinement during study delivery [64]. Sample size might also depend on the richness of the data, and, in studies where rich sampling is usually not possible (e.g. neonatal studies), optimal design can prove useful by providing the most informative time points for sampling, thus reducing the number of samples required per subject enrolled [65]. Using optimal design to identify the most informative sampling times in each pediatric age group can, at the same time, also help to increase the power of the PKPD analysis.

## Rationale for Using Modeling

There are numerous advantages to using a model-based approach over the (traditional) data summary approach (i.e. calculate AUC, *C*_max_, etc) for PK or PD studies in children [66]. For example, non-linear mixed-effects modeling facilitates the analysis of sparse, unbalanced datasets, which are common in neonatal and pediatric research settings, where each individual may only contribute a small number of samples, and sample timing/number of samples can vary between patients—as, for example, in studies with opportunistic sampling [23, 67, 68]. Furthermore, models can incorporate factor-relevant covariates, such as age and weight, which enable us to evaluate the developmental differences between adults and children, in addition to ontogeny and pharmacogenetic factors [23]. The developed model can also account for the whole concentration–time course, and hence can readily be used as a link with the effect (PD).

A detailed description of the statistical and mathematical aspects of PKPD modeling is beyond the scope of this review, but a brief summary of typical modeling methods for analyzing PKPD data is provided in Table 2 [67].

**Table 2:** Overview of core pharmacokinetic analytical methods [67]

Method	Description	Comments
Naive pooled data approach	All PK data from the study are pooled and analyzed as if from one individual	The analysis does not incorporate the fact that the data arise from individuals with between-subject variability, and can give biased parameter estimates; it can be used in unbalanced study designs but will overestimate variability and can lead to biased parameter estimates
Naive average data approach	The mean drug concentration at each time point in the PK study is calculated, based on the data at that time point contributed by all participants. The mean value at each sampling time is then used to estimate the PK parameters of interest	This simplistic approach is popular but is unreliable and limited because it does not consider inter- or intraindividual variability, and therefore underestimates variability. It is only suitable for a balanced study design
Two-stage approach	The PK parameters are first estimated for each individual, then the variance of these parameter estimates is calculated	This method is attractive because it is mathematically straightforward, but requires rich individual-level data
Non-linear mixed effect modeling (NLME)	All study data are fitted simultaneously in one model, but the PK parameters are able to vary between individuals	This approach has become standard practice because it provides unbiased parameter estimates through simultaneous quantification of parameter-level interindividual variability, and observation-level residual variability

*PK* pharmacokinetic

## Specific Issues in Pediatric Pharmacokinetic/Pharmacodynamic (PKPD) Modeling

### Scaling of Pharmacokinetics

#### Different Approaches for Scaling Pharmacokinetics

Children, especially infants, are smaller, weigh less, and have a higher proportion of total body water and lower proportion of body fat compared with adults. Furthermore, key organ function, specifically kidney and liver function, is immature in newborns and infants, resulting in a lower glomerular filtration rate (GFR) and a distinct hepatic enzyme activity profile. Membrane-bound drug transporters also vary with age [69]. These numerous physiological differences between adults and children, particularly infants, contribute to PK processes, and consequent age- and size-related differences in PK parameters [70]. Therefore, PK parameters cannot simply be scaled linearly from adults to children; instead, approaches such as allometric scaling, physiologically-based pharmacokinetic (PBPK) models [71], and systems pharmacology modeling [72] should be used [73].

PBPK models are represented by a complex system of equations with parameters that incorporate biological knowledge of physiological blood flows, anatomical organ structures, and also tissue and organ volumes [74]. PBPK models can be used to predict first-in-man drug doses (prior to human exposure) [75], first-in-children doses [76], and, more recently, for drug–drug interaction studies [77], but have also been suggested for scaling PK to children [78, 79]. If the aim of a PBPK model is extrapolation to the pediatric population, it must either firstly describe the adult data well and include data on all developmental changes affecting drug pharmacokinetics [80, 81], or, alternatively, an existing PBPK model for children of a certain age and medical condition can be used, together with the physicochemical properties of the studied drug. However, extrapolating across all age groups might be difficult for some physiological spaces, for example brain and bone marrow [80]. It can also be challenging to obtain sufficient in vitro data for some compounds, which could then lead to poor predictive performance [82]. Some authors have fitted PBPK models to PK data to overcome this [83, 84]; however, this approach is not yet widely used in pediatric research, but is growing in application and can be used to update or refine the PBPK model. Although the validation requirements for PBPK models are not yet as well defined as, for example, those for population PKPD modeling, the average fold error is often used as a guiding metric for validation [85, 86].

Systems pharmacology models are based on a network of nodes (or functional elements), with functional interactions between them. Recently, these models have been suggested to be useful for describing disease progression and complex drug action patterns [72].

An alternative to extrapolating PK from adults to children is to perform model-based meta-analysis of existing pediatric data [73], or an empirical analysis of the observed pediatric drug PK data [73], where characteristics of patients can be used as covariates to help explain and describe the ontogeny of a PK parameter. These two approaches are especially useful when, for example, designing a new clinical trial (to e.g. test a new dosing regimen) in a similar pediatric population. Since pharmacological effects of many drugs are driven by drug exposure (AUC), which is indirectly proportional to CL, we usually focus on scaling CL. As CL changes as humans grow and age/mature, models need to account for these two, correlated processes.

For drugs that are orally administered, the influences of age-appropriate formulations and developmental differences in bioavailability on pharmacokinetics also need to be considered, which are discussed in detail elsewhere [87]; these factors can also be taken into account with model-based approaches to investigate pediatric PK.

#### Body Size

Almost 70 years ago, Crawford et al. noted that using body surface area (BSA) is preferred over linear weight for predicting doses [88]. Decades later, it was suggested that a so-called allometric approach (Eq. 1), which scales metabolic processes with body size, could also be used to explain changes in drug CL [89]:

1 $$y_{i} = a \cdot {\text{WT}}_{i}^{b} ,$$

where *y*_*i*_ is the individual subject’s body function of interest (that is being predicted), WT is the individual’s body weight in kilograms, *a* is the allometric constant, which assumes the value of *y* when WT = 1 kg, and *b* is the allometric exponent (*b* < 1 indicates that the body function increases slower with body size than weight). Hereafter, we use the term allometric scaling to refer to allometric scaling of CL.

Historically, although studied for almost a century, there has been no agreement on which value of the allometric exponent to use, but generally values between 0.63 and 0.78 have been suggested. For example, in the 1930s, Benedict proposed the use of 0.67 since it was found that the basal metabolic rates scale best with BSA, which is approximately WT^0.67^ [90]. Around the same time, Kleiber looked at 13 different species of mammals with a wide weight range (0.15–679 kg) and concluded that the value of the allometric exponent should be 0.75 [91]. However, he also noticed that the value of the exponent (0.67 or 0.75) only altered predictions if the difference between the subjects’ weight was at least ninefold [92]. More recently, GFR was found to scale with weight raised to the power of 0.63 [93], and hepatic blood flow with WT^0.78^ [94]. In a recent meta-analysis of almost 500 PK studies, McLeay et al. [95] found that, when estimated, the median value of the allometric exponent was 0.65 (range − 1.2 to 2.2), but the most common fixed value was 0.75 (also most often used in pediatric PK studies). Although the allometric exponent remains a highly controversial topic, the use of a fixed allometric exponent of 0.75 (combined with a maturation function for younger children) was also supported by our recent study, which compared 18 approaches for scaling CL [96].

#### Size and Maturation

Using allometric models alone, which only account for size-related CL changes, is not sufficient [79, 97], particularly for neonates and infants, since CL is frequently lower than expected in these pediatric populations due to the physiological immaturity of their organs [98]. Therefore, age also needs to be taken into account, especially when analyzing data from neonates as their organ functions change very rapidly [70]. Taking both size and age into account in model development can help capture CL changes across the whole pediatric age range.

There has been much heated debate about how best to account for both size and maturation in pediatric PK studies [71, 82, 99]. While we know that “all models are wrong, some are useful” [100], a wide range of different approaches can impede our ability to compare parameters between studies of the same (or similar) drugs, by creating added complexity. Importantly, using a standardized method for parameterizing size and age across studies could aid extrapolation, improve study design, and potentially allow for smaller sample sizes. In a recently published paper, we identified the various approaches taken to scaling CL, and provided a direct comparison of these methods using the same dataset for two drugs with different routes of elimination, specifically glomerular filtration and hepatic metabolism [96]. In light of the results, and in the knowledge that weight and age are highly correlated, albeit with the correlation varying at different ages, we recommend using a combination of allometric weight scaling with a sigmoidal maturation function (Eq. 2) to describe the changes in CL due to age and weight. By adding age into the model one can estimate the deviation (especially in the younger group) from CL, predicted using only the biological prior knowledge of allometric scaling (i.e. size).

2 $${\text{CL}}_{\text{child}} = {\text{CL}}_{\text{adult}} \cdot \left( {\frac{{{\text{WT}}_{\text{child}} }}{70}} \right)^{b} \cdot \frac{{{\text{PMA}}^{\text{Hill}} }}{{{\text{PMA}}_{ 5 0}^{\text{Hill}} {\text{ + PMA}}^{\text{Hill}} }},$$

where CL_child_ is the predicted drug CL for a studied child, CL_adult_ is the typical CL for a 70 kg adult, *b* is the allometric exponent that can be estimated, but fixing to 0.75 is advocated (especially if the WT range in the studied population is small), PMA is the child’s postmenstrual age (usually in weeks), PMA_50_ is the PMA when CL has reached 50% mature, and Hill is the sigmoidicity/shape parameter.

Using postmenstrual age to account for preterm neonates is important, although it should be noted that additional postnatal age scaling may be needed due to physiological changes at birth regardless of gestational age [101]. Additionally, it is sometimes also necessary to add a so-called organ function, accounting for the difference in the organ function between healthy and diseased; for example, a function including serum creatinine concentration in the case of a renally excreted drug (or another suitable biomarker reflecting renal function) [8, 101].

Including a standardized parameterization for age and size in PKPD models reported in publications would enable comparison of parameters across studies of the same or similar compounds. When planning new studies, drug-, organ- or enzyme-specific maturation models can be used in the prediction of expected CL to a similar pediatric population. Such models can also be used when fitting data, and literature models may be particularly useful in this context when small age ranges are studied [101–103]. In this case, the maturation model may be fixed or introduced as a Bayesian or ‘frequentist’ prior. This approach to extrapolation requires further research and is likely to be limited by drug physicochemical properties.

### Dose Selection in Pediatric Studies: Extrapolation and Prediction

Determining a first pediatric dose is difficult because one needs to take into account efficacy as well as safety since it would usually not be considered ethical to give a child an ineffective dose (microdosing studies excepted [104]). Ideally, a PK study in the pediatric population would be used to define a pediatric dose, but this is not always possible [82, 105]. The pediatric dose is thus usually predicted by down-extrapolating the adult dose [106]. Historically, this has been done with empirical methods: linear weight scaling or non-linear allometric weight scaling (Eq. 3) [8, 107]:

3 $${\text{Dose}}_{\text{child}} {\text{ = Dose}}_{\text{adult}} \cdot \left( {\frac{{{\text{WT}}_{\text{child}} }}{ 7 0}} \right)^{b} ,$$

where WT_child_ is the weight of a child in kilograms. No single value of *b* is suitable across the whole pediatric age range [82, 106], without also accounting for maturation [108]. Scaling CL linearly with weight from adults to neonates while ignoring maturation can lead to serious adverse reactions, such as ‘gray baby syndrome’ [109], and kernicterus [110], which occurred after the administration of chloramphenicol and sulphonamides to infants.

Thus, now that scaling for both size and maturation is well established (as outlined above—although there may be a time lag before it is widely adopted), a more sophisticated approach can be used to account for the rapid increase in weight and the concurrent changes in organ function maturation. Instead of scaling the dose directly, we can scale PK in order to obtain pediatric PK parameter estimates and determine the dose for a child of any age or weight, without the need for arbitrary cut-off values for either covariate (as typically specified in the non-standardized formulae for scaling CL) [8]. As mentioned above, there are strong arguments to advocate that adult CL should ideally be scaled to children using a standardized approach [96]. Using a model-based approach to guide dosing in children has previously been suggested [111, 112], and efforts to develop and validate individualized dosing software are ongoing in both children [113] and adults [114]. It should be noted that caution is required when extrapolating across populations if the covariates within the target population lie outside the range included within the model development dataset as parameter–covariate relationships can change in different pediatric populations [47]. In such instances, where this caveat is unavoidable, it may be helpful to combine ‘bottom-up’ approaches (e.g. PBPK) and ‘top-down’ approaches (e.g. population PK) to first test whether the different methods produce reasonably similar results [115].

Obese children may also need to have their dose adjusted for certain agents [116], with lean body weight being suggested as the preferred body size descriptor in this population [117, 118]. Another possible size descriptor could be fat-free mass (FFM). A model for predicting FFM from a child’s weight, age, sex, and body mass index (BMI) has recently been developed [119]; however, this model only included data from children > 3 years of age, and therefore cannot be used for neonates and infants. Furthermore, BMI is unsuitable for use in neonates. More studies focusing on obese and overweight children are needed before the best body size descriptor can be defined and before concluding whether dose adjustment is clinically necessary [116, 117].

Standardized parameterization for the scaling of CL in pediatric PK models is likely to remain a topic of some controversy, but, in light of the numerous benefits it confers, we believe its importance is paramount. However, we are not suggesting that pharmacokinetic modeling analyses should employ only this method in isolation, but would instead encourage analysts to publish their results using the standardized parameterization, in addition to the results using other parameterizations that they identify as providing a good fit to the data. This will enable ongoing collation of data about the performance of this method for drugs with different physicochemical properties and distinct pharmacokinetic profiles.

### Scaling of Pharmacodynamics

While much recent emphasis has been placed on scaling PK, methods for investigating maturation and scaling of pediatric PD have frequently been neglected. This might be attributed to the fact that drug effect is more difficult to measure and evaluate, especially in neonates and infants [8]. PD endpoints can vary widely depending on the disease, therefore there will never be a single, unified method for PD scaling. For example, the GABAergic inhibitory system within the central nervous system is immature in neonates compared with adults, causing benzodiazepines to paradoxically exacerbate seizures, especially in premature newborns [120, 121]. With reference to the immune system, thymic output of T cells is higher in children compared with adults, with a peak in the thymic output at approximately 1 year of age [122]. Advanced knowledge of PD maturation might be anticipated in anesthesia, where drug effect is monitored in real time. It has long been known that for inhalational anesthetics, the alveolar concentration required for 50% of patients not to react to surgical incision initially rises, then falls with age [123]. In contrast, for propofol, Peeters et al. found that the target concentration in infants is the same as in adults, and dose differences were due to PK maturation [124]. In view of the extensive differences in PD between adults, children, and neonates, a greater use of PD modeling—rather than just simple extrapolation—is needed between these populations in order to reach and verify an efficacy target equivalent to adults [125].

### Model Evaluation

A model should always be evaluated before it can be used for extrapolation [126]; several internal and external validation methods can be employed for this purpose. Internal methods include diagnostic goodness-of-fit plots, such as observed values of dependent variables plotted against population and/or individual predicted values [127, 128]. The distribution of the residual errors should also be examined to confirm whether they are normally distributed; conditional weighted residual errors can be plotted versus time or versus the population predictions to test this assumption. In addition to prediction-based evaluation, methods can also be (Monte Carlo) simulation-based, including, for example, visual predictive checks (VPCs), and plots investigating whether the distribution of normalized prediction discrepancies (NPD) follows a normal distribution [128]. Superior to internal evaluation is external evaluation, where the ability of a model to predict data that were not used for model building is assessed. When a separate dataset for external evaluation is not available, a so-called cross-validation approach can be used instead, where a dataset is split several times into a model building and model evaluation dataset, and then, for example, prediction errors are calculated [126].

## Role of PKPD Modeling in Pediatric Trial Design

PKPD modeling can be applied to various further aspects of study design. For example, as briefly introduced above, it can be used in the context of optimal design to identify the most informative sampling schedule, number of samples per participant, and sample size; specialist software is available to support these processes [129–132]. Optimal design methods and concepts applied to pediatric PKPD studies have recently been reviewed by Roberts et al. [133]. When designing PKPD trials it is essential to account for patient acceptability and logistical factors in running the trial. Simple designs may be preferable, where feasible, providing they are scientifically sound [134], although caution should be exercised when employing opportunistic sampling to ensure methodological suitability for the drugs/analytes being studied [135, 136]. PKPD modeling can also incorporate knowledge regarding the expected placebo effect (where relevant) and anticipated rates of study attrition [137, 138]. Early engagement with children and families (patient–public involvement) to help guide decision making can provide valuable input to guide study design and research ethics committees (Institutional Review Boards) when reviewing protocols [139]. These considerations are particularly important in certain settings, such as pediatric/neonatal intensive care, oncology, or resource-limited environments, and the relevant context-specific, ethical, and practical issues should be factored into study design [140–143].

## Role of PKPD Modeling in Dose Optimization Strategies

Once drugs are in routine clinical use in pediatric patients, there are further applications of PKPD modeling, which have also been developing rapidly in recent years. As mentioned above, model-based decision support strategies can be used to guide TDM or dose individualization approaches in patient populations where pharmacokinetic variability is clinically important [101, 114, 144–150]. At present, the availability of the necessary software and expertise is highly variable, even in resource-rich settings, but it is anticipated that these techniques will be more frequently used in the clinic over the next 5–10 years. Should adaptive licensing become commonplace in future [103, 151, 152], then the importance of these dose optimization concepts will be further reinforced.

## Moving Forward in this Field

The importance of PKPD modeling in pediatric drug development continues to grow, and it will clearly have a pivotal role in clinical pharmacology research throughout the lifecycle of medicinal products in the twenty-first century. Ensuring relevant stakeholders in drug development are familiar with the central tenets in PKPD modeling will enhance successful applications of these methods to improve efficiency in the drug development pipeline.

The scaling principles discussed in this review are equally applicable to small molecules and biologics [153]. Initiatives to aid data and model warehousing, such as the Drug Disease Model Resources (DDMoRe) collaboration [154, 155], and standardization of trial conduct, reporting and analysis methods [156–158], will hopefully lead to greater potential for learning across compounds.

Growing recognition that personalized medicine incorporates all aspects of variability (including genetics) will see expanding use of model-based TDM. There is also a clear need to engage with the gene therapy clinical trials community because the mathematics and statistics of dosing regimen design, and assessing treatment impact on disease progression, which have developed in clinical pharmacology, are also highly relevant in this context.

## Conclusions

PKPD modeling will remain critically important in the design and conduct of clinical pharmacology research in children, particularly during drug development. Pediatric PK has now moved on from the paradigm ‘children are not small adults’ towards recognition of how important parameters scale with both size and age. Once appropriate functions for size and age are added to a PK model, this allows their effects to be delineated from other patient-specific factors such as severity of disease state and organ dysfunction. Instead of using complicated methods for scaling, which risks delivering parameters that are difficult to interpret, we advocate one standard approach for pediatric scaling of CL using a combination of allometric weight scaling to account for size-related changes in CL, and a sigmoid function to describe age-related maturation of CL. When parameterization for age and size is standardized, comparison of parameters across studies of the same or similar compounds is readily enabled. Due to the heterogeneity of PD endpoints and regulatory guidance allowing PK-only extrapolation in some cases, much less attention has been paid to the scaling and maturation of drug effects to date. Since PD endpoints are often easier to collect than PK [152, 159], it is now time that the assumptions of equal efficacy with similar exposure are challenged in all age groups using PKPD modeling in order to progress towards the ultimate goal of truly optimized pediatric pharmacotherapy.
