# Correction to: Pharmacokinetic–Pharmacodynamic Modeling in Pediatric Drug Development, and the Importance of Standardized Scaling of Clearance

**DOI:** 10.1007/s40262-018-0723-9

**Published:** 2018-12-17

**Authors:** Eva Germovsek, Charlotte I. S. Barker, Mike Sharland, Joseph F. Standing

**Affiliations:** 1 Infection, Inflammation and Rheumatology Section, UCL Great Ormond Street Institute of Child Heath, University College London, London, UK; 2 Pharmacometrics Research Group, Department of Pharmaceutical Biosciences, Uppsala University, PO Box 591, 751 24 Uppsala, Sweden; 3 Paediatric Infectious Diseases Research Group, Institute for Infection and Immunity, St George’s, University of London, London, UK; 4 St George’s University Hospitals NHS Foundation Trust, London, UK

## Correction to: Clin Pharmacokinet 10.1007/s40262-018-0659-0

The Open Access license, which previously read:

**Open Access** This article is distributed under the terms of the Creative Commons Attribution-NonCommercial 4.0 International License (http://creativecommons.org/licenses/by-nc/4.0/), which permits any noncommercial use, distribution, and reproduction in any medium, provided you give appropriate credit to the original author(s) and the source, provide a link to the Creative Commons license, and indicate if changes were made.

Should read:

**Open Access** This article is distributed under the terms of the Creative Commons Attribution 4.0 International License (http://creativecommons.org/licenses/by/4.0/), which permits unrestricted use, distribution, and reproduction in any medium, provided you give appropriate credit to the original author(s) and the source, provide a link to the Creative Commons license, and indicate if changes were made.

